# In for the long-haul? Seropositivity and sequelae 1 year post COVID-19

**DOI:** 10.1186/s12916-021-02084-4

**Published:** 2021-09-14

**Authors:** Jeremy Werner Deuel, Patricia Schlagenhauf

**Affiliations:** 1 Epidemiology, Biostatistics and Prevention Institute, WHO Collaborating Centre for Travellers’ Health, University of Zurich, Zurich, Switzerland; 2 Competence Centre for Military Medicine and Biology, Hirschengraben 84, 8001 Zurich, Switzerland; 3 Wellcome-MRC Cambridge Stem Cell Institute, University of Cambridge, Puddicombe Way, Cambridge, CB2 0AW UK

**Keywords:** COVID-19, Long-COVID, SARS-CoV-2

## Background

Health care workers (HCW) who have been on the front-line of the pandemic, highly exposed to SARS-CoV-2 and frequently infected, are now the focus of research on the longer-term evolution of COVID-19. This is important as HCWs are generally young, previously healthy adults and are representative of the active “workforce” in countries worldwide. An infection with SARS-CoV-2 can be seen as a twin-edged sword: on one hand, having had the infection will confer some immunity or protection against re-infection whilst on the other hand, infection sequelae may result in subtle or significant health consequences. How long will the infection -conferred, antibody reactivity to the receptor-binding domain of SARS-CoV-2, the neutralising activity and the specific memory B cells last post infection? Mounting evidence suggests that this protection may last for 12 months or longer [1]. What is the price of this COVID armour? The cost is steep!

SARS-CoV-2 infected individuals may experience debilitating sequelae that persist for months and longer. A systematic review on long-term sequelae in previously healthy, young people aged < 50 years old found a broad spectrum of symptoms [2]. These included persistent fatigue, breathlessness, diminished quality of life, impaired pulmonary function, myocarditis, neurological and psychiatric diagnoses, slow and stagnant recovery of olfactory and gustatory function. Due to the global proliferation of COVID-19 infection, the burden of sequelae is thus projected to be immense.

## Long-lasting immunity after COVID-19 in health care workers

In this edition of *BMC Medicine*, three papers are presented showing a one-year follow up after COVID-19. Dobaño et al. [3] followed up a cohort of 173 Spanish health care workers one year after COVID-19. More than 90% remained seropositive and only four re-infections were reported. This suggests a long-lasting immunity, that is protective, with respect to re-infection for the vast majority of convalescents; however, the few low- or non-responders seem to be at risk for a second symptomatic infection with COVID-19. These findings are reassuring but also raise the question as to whether a year or more, post-infection, will the neutralising activity also work as well against the Variants of Concern (VoCs) as against the wild-type SARS-CoV-2 virus? A recent analysis showed that people who were SARS-CoV-2 infected and vaccinated had higher plasma antibodies and nearly a fiftyfold increase in neutralising activity compared to those who were previously infected but unvaccinated [1]. This has major implications for HCW who have been infected during the earlier phase of the pandemic and who may now be exposed to variants for concern. Vaccinate post infection!

## Long-COVID in health care workers

Besides long-lasting immunity, COVID-19 also leads to long-lasting sequelae, now termed long-COVID [2]. Xiong et al. [4] follow up a cohort of 333 health care workers (HCW) with severe COVID-19 and a median age of only 36 years after 5, 8, and 11 months. Importantly, nearly one third of these showed persistent symptoms of long-COVID 1 year after COVID-19 with a decline in muscular strength, flexibility as well as agility and dynamic balance. Similar results have been reported one month after COVID-19 in a cohort of young Swiss soldiers [5]. The authors of this study also reported detectable antibody titers in more than 90% of the infected one year after COVID-19. Importantly, inflammatory cytokines such as TNF-alpha or IL-6 were measured in this cohort and seem to decay slowly. The authors report an increase in IgM titers, which could be indicative of (continuous?) re-infection of HCW, although more data are needed to answer this question.

## Long-lasting functional and structural lung changes after COVID-19

Chen et al. [6] followed up a cohort of 41 patients post COVID-19 with a median age of 51 years using repetitive CT imaging and lung function testing. 47% showed residual chest CT changes one year after discharge with age being the main risk factor for such residual changes. After six months, one third of the patients had remaining changes in the lung CT (ground-glass opacities and reticular pattern) and one fifth of the patients had an abnormal CO-Diffusion or total lung capacity of more than a year after COVID-19. These observations are compatible with a fibrotic process in the lung but are no definitive proof thereof, since no biopsies were performed. The authors nicely show the dynamics of CT graphic changes with a peak three weeks after onset of symptoms and a slow decline over the following months.

## Conclusions

Larger, systematic studies quantifying overt and subtle COVID sequelae based on objective criteria as well as assessing the persistence and robustness of immunity are needed. Such studies should ideally include a COVID-19 naive control group as well as asymptomatically infected and symptomatic COVID-19 convalescents. Because “long-COVID” is projected to impact several body systems, test batteries must be comprehensive, quantitative and sensitive enough to detect subtle differences between such groups and to adequately define the disability associated with sequelae of the infection. With projected millions of “long haulers” in the workforce, investigators will need to consider using non-invasive testing, organ damage biomarkers and sample biobanking to elucidate the extent of the post-pandemic impact of COVID-19 and define the burden of long-COVID. Such studies are already in progress [7] and may detect hitherto unknown, long-term sequelae, and give insight into the pathophysiology and consequences of SARS-CoV-2 infection. Such data are urgently needed to prepare for the societal impact of COVID-related workforce disability and to develop strategies to mitigate these sequelae. One thing is certain, the long haul has just begun.

## Data Availability

Not applicable
